# Reduced MLH3 Expression in the Syndrome of Gan-Shen Yin Deficiency in Patients with Different Diseases

**DOI:** 10.1155/2017/4109828

**Published:** 2017-09-26

**Authors:** Juan Du, Maofeng Zhong, Dong Liu, Shufang Liang, Xiaolin Liu, Binbin Cheng, Yani Zhang, Zifei Yin, Yuan Wang, Changquan Ling

**Affiliations:** ^1^Department of Chinese Medicine, Changhai Hospital, Second Military Medical University, Shanghai 200433, China; ^2^Shanghai University of Traditional Chinese Medicine, Shanghai 201203, China; ^3^E-Institute of TCM Internal Medicine, Shanghai Municipal Education Commission, Shanghai 201203, China

## Abstract

Traditional Chinese medicine formulates treatment according to body constitution (BC) differentiation. Different constitutions have specific metabolic characteristics and different susceptibility to certain diseases. This study aimed to assess the characteristic genes of gan-shen Yin deficiency constitution in different diseases. Fifty primary liver cancer (PLC) patients, 94 hypertension (HBP) patients, and 100 diabetes mellitus (DM) patients were enrolled and classified into gan-shen Yin deficiency group and non-gan-shen Yin deficiency group according to the body constitution questionnaire to assess the clinical manifestation of patients. The mRNA expressions of 17 genes in PLC patients with gan-shen Yin deficiency were different from those without gan-shen Yin deficiency. However, considering all patients with PLC, HBP, and DM, only MLH3 was significantly lower in gan-shen Yin deficiency group than that in non-gen-shen Yin deficiency. By ROC analysis, the relationship between MLH3 and gan-shen Yin deficiency constitution was confirmed. Treatment of MLH3 (−/− and −/+) mice with Liuweidihuang wan, classical prescriptions for Yin deficiency, partly ameliorates the body constitution of Yin deficiency in MLH3 (−/+) mice, but not in MLH3 (−/−) mice. MLH3 might be one of material bases of gan-shen Yin deficiency constitution.

## 1. Introduction

The foundation of clinical Traditional Chinese Medicine (TCM) practice was dependent on the syndrome differentiation theory but not the concept of disease in modern medicine [1]. Syndrome is a summarization of the pathological changes of a disease at a certain stage in its course of development. The theory of Yin-Yang is the principle of syndrome differentiation. The occurrence, development, and changes of disease, according to the theory of Yin and Yang, lie in the imbalance between Yin and Yang. The imbalance of Yin and Yang includes excessiveness or deficiency of Yin and Yang. Gan-shen Yin deficiency, one of the major subtypes of Yin deficiency, is very common in primary liver cancer (PLC), diabetes mellitus (DM), and high blood pressure (HBP) patients, which express emaciation, soreness and weakness of the lumbar region, night sweat, dizziness, syrigmus, and so on. However, exactly what is gan-shen Yin deficiency and if there are same pathological status and basis materials in patients with different diseases are still unclear. The syndrome research is suffering the question of credibility because of the lack of experimental data support.

The material basis of TCM syndrome in physiological functions and pathological status had been studied using clinical and animal experiments in recent years. Because characteristics of genes expression, regulation, and production are closely similar to TCM philosophy, the relationship between genes and material basis of TCM syndrome may be a potential research direction.

In our previous study, there are seventeen characteristic mRNA variables in PBMCs of gan-shen Yin deficiency patients with PLC using microarray analysis [2]. To confirm the results, the 17 characteristic mRNA variables were further determined in gan-shen Yin deficiency patients with different disease including PLC, HBP, and DM and screened for factors significantly associated with gan-shen Yin deficiency syndrome. Then Liuweidihuang wan is used, which is a kind of classic therapeutic drugs for gan-shen Yin deficiency syndrome, to confirm the potential biomarker in genetically modified mice and provide scientific information for the gene's roles in the treatment of gan-shen Yin deficiency syndrome.

## 2. Materials and Methods

### 2.1. Subjects

The study participants were men and women over 18 years old and were recruited between January 2013 and February 2015 from the TCM department, endocrinology department, and cardiology department of Changhai Hospital, the endocrinology department and cardiology department of Shanghai Traditional Chinese Medicine Hospital, and Shanghai Shuguang Hospital (Shanghai, China). Diagnosis and staging standards of PLC referred to “the standard of clinical diagnosis and staging of primary liver cancer” (Chinese Society of Liver Cancer, 2001) [3]. Diagnosis and staging standards of DM referred to “the criteria of the American Diabetes Association.” Diagnosis and staging standards of HBP referred to “Chinese Guidelines for the Management of Hypertension in the Community (2016 revised edition).” This study complied with the Declaration of Helsinki. The relevant institutional ethics review boards approved the protocol, and all participants provided informed consent.

A Chinese medical doctor, Juan Du (female aged 38), with a degree in medicine, a license as a Chinese physician, and more than 7 years of clinical experience, explained the details of the study to the subjects and solicited written informed consent from each subject. Patients who had taken Chinese medicine within the past month were excluded from the study. A total of 244 subjects (50 PLC patients, 94 HBP patients, and 100 DM patients) were recruited. The characteristics of the participants are shown in Table 1.

### 2.2. Design and Study Population

Three types of data were collected including the body constitution questionnaire (BCQ) responses, the stage of the disease, and clinical records of a Chinese medical doctor (CMD). The BC was assessed from the self-reported BCQ responses and by the CMD. Clinical manifestations of gan-shen Yin deficiency and non-gan-shen Yin deficiency were evaluated using the BCQ [4, 5], while the CMD reviewed the results of each indicator of gan-shen Yin deficiency and non-gan-shen Yin deficiency. In order to ensure the quality of investigation, three important steps were taken throughout the whole process of the investigation. First of all, all potential participants were approached by a Chinese medicine professional who gave them an introduction on TCM constitution first; then a well-trained research staff gave them a brief introduction of the study. Written informed consent to participation was obtained prior to data collection which was carried out via self-administered questionnaire as well as face-to-face consultation. Finally, the interviewer checked the questionnaire so as to complement the missing data and verify the illogical data as much as possible. Secondly, to ensure consistency of the survey across sites and over time, all patients were investigated using the same procedures and standards by the same interviewer to ensure interexaminer reliability. Thirdly, to ensure the TCM syndrome differentiation was not affected by the disease, all patients with PLC, DM, and HBP in two groups were distributed in the same stage of the disease, separately.

Finally, 25 PLC patients, 44 HBP patients, and 50 DM patients were included in gan-shen Yin deficiency group and another 25 PLC patients, 50 HBP patients, and 50 DM patients were included in non-gan-shen Yin deficiency group according to the BCQ results. The anticoagulated blood (5 ml) was collected from all patients between 6:30 and 7:00 a.m. All the blood samples were used for PBMC isolation.

### 2.3. Real-Time RT-PCR

Seventeen biomarkers were selected and analyzed for discriminate gan-shen Yin deficiency from other constitutions using real-time RT-PCR [6].

### 2.4. Animals

Seven-week-old male MLH3 (−/+ and −/−) mice were obtained from the Jackson Laboratories (Bar Harbor, ME, USA). C57BL/6 wild-type (WT) mice were obtained from Southern Model Biological Technology Development Co., Ltd. (Shanghai, China). MLH3 (−/+) mice were used to establish the H22 tumor bearing model, HBP model, and diabetes model. H22 tumor bearing mice were established as [7]. Hypertension was induced by being fed with HSD (8% NaCl with tap water at 1% of NaCl) during a period of 8–18 weeks [8]. Diabetes was induced with a single intraperitoneal injection of streptozotocin (STZ) (Sigma, Madrid) 200 mg/kg of body weight, freshly dissolved in citrate buffer (pH = 4.5) [9]. All animals (MLH3 −/− and −/+ mice) were divided into control group and Liuweidihuang wan treatment group, which was administered orally with Liuweidihuang wan (10 g/kg) for 2 weeks, a kind of famous prescription for gan-shen Yin deficiency.

Animal experiments were approved by the Animal Care and Use Committee of Second Military Medicine University and conformed to the Guide for the Care and Use of Laboratory Animals (NIH, Bethesda, MD, USA).

### 2.5. ELISA

The levels of E_2_, T in serum, and cAMP in plasma were determined by ELISA [6].

### 2.6. Data Analyses

Statistical analyses were performed using SPSS version 19.0 (SPSS, Chicago, IL). Data were analyzed when appropriate by using simple descriptive analyses, such as mean ± standard deviation ($\overset{-}{\chi }\pm S$) and ranked data. Wilcoxon rank sum test, *χ*^2^ test, and Fisher's exact tests (whenever appropriate) were used to explore the differences of the variables. The predictive accuracy was calculated using the ROC. All reported *P* values were two sided, and statistical significance level (*α*) was set at 0.05.

## 3. Results

### 3.1. Comparisons of Basic Characteristics between Constitutions of Gan-Shen Yin Deficiency and Non-Gan-Shen Yin Deficiency

The study group was composed of 50 PLC patients, 94 HBP patients, and 100 type 2 diabetics. Of the 244 participants, 119 were categorized as gan-shen Yin deficiency syndrome characterized by soreness and weakness of the lumbar region, night sweat, dizziness, syrigmus, and so on, and others were categorized as non-gan-shen Yin deficiency syndrome according to BCQ. Table 1 compares the age, gender, and chronic disease conditions between patients with and without gan-shen Yin deficiency. The BMI in the gan-shen Yin deficiency patients with DM was significantly lower than that in non-gan-shen Yin deficiency group (*p* < 0.05). No other significant difference was found between two groups.

### 3.2. Analysis on the Differentially Expressed Genes Associated with Gan-Shen Yin Deficiency Syndrome in Patients with Different Diseases

**Figure 1:**
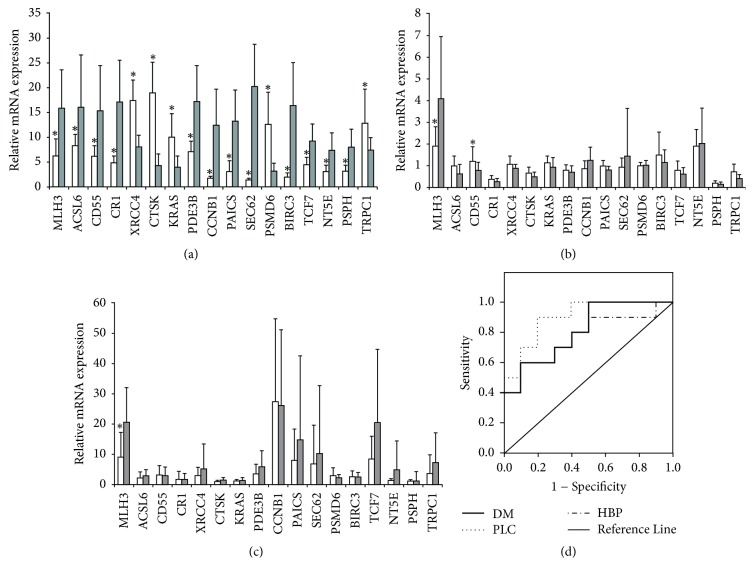
Analysis on the differentially expressed genes associated with gan-shen Yin deficiency in patients with different diseases. (a) Real-time RT-PCR analysis of 17 mRNAs in PLC patients with or without gan-shen Yin deficiency symptoms. (b) Real-time RT-PCR analysis of 17 mRNAs in HBP patients with or without gan-shen Yin deficiency symptoms. (c) Real-time RT-PCR analysis of 17 mRNAs in DM patients with or without gan-shen Yin deficiency symptoms. ^*∗*^*p* < 0.05 compared with non-gan-shen Yin deficiency group. (d) ROC curve in MLH3 expression to calculate the best cutoff values to discriminate between LPC patients with or without gan-shen Yin deficiency symptoms.

The seventeen characteristic mRNA variables in PBMCs of PLC patients with gan-shen Yin deficiency syndrome were further determined. To explore the genes most closely related to gan-shen Yin deficiency, we measured the transcriptional expression of these 17 mRNA variables in 25 PLC samples, 44 HBP samples and 50 DM samples from gan-shen Yin deficiency groups and 25 PLC samples, 50 HBP samples, and 50 DM samples from non-gan-shen Yin deficiency groups. Of the PLC samples, we found that the transcriptional expressions of* MLH3, ACSL6, CD55, CR1, PDE3B, CCNB1, PAICS, SEC62, BIRC3, TCF7, NT5E,* and* PSPH* in the PBMCs of gan-shen Yin deficiency group were significantly lower than those in non-gan-shen Yin deficiency group (Figure 1(a), *p* < 0.05). The transcriptional expressions of* KRAS, TRCP1, CTSK, PSMD6,* and* XRCC4* were significantly higher than those in non-gan-shen Yin deficiency group (Figure 1(a), *p* < 0.05).

Of the HBP samples, we found that the transcriptional expression of MLH3 in the PBMCs of gan-shen Yin deficiency group was significantly lower than that in non-gan-shen Yin deficiency group (Figure 1(b), *p* < 0.05). The transcriptional expression of CD55 was significantly higher than that in non-gan-shen Yin deficiency group (Figure 1(b), *p* < 0.05).

Of the DM samples, we found that the transcriptional expression of MLH3 in the PBMCs of gan-shen Yin deficiency group was significantly lower than that in non-gan-shen Yin deficiency group (Figure 1(c), *p* < 0.05).

From ROC analysis, we found that 90.0% of the PLC patients, 77.0% of the HBP patients, and 81.0% of the DM patients with gan-shen Yin deficiency syndrome had low expression of MLH3 (Figure 1(d), AUC = 0.90, *p* = 0.002; AUC = 0.77, *p* = 0.041; and AUC = 0.81, *p* = 0.019).

### 3.3. MLH3 KO Mice Were Used to Validate the Status of MLH3 in Gan-Shen Yin Deficiency Syndrome

**Figure 2:**
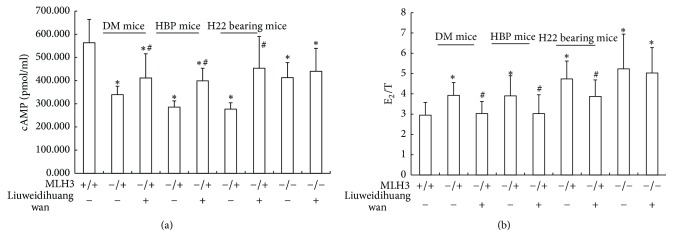
MLH3 KO mice were used to validate the status of MLH3 in gan-shen Yin deficiency. (a) ELISA analysis of cAMP in plasma of mice. (b) ELISA analysis of E_2_ and T in serum of mice. ^*∗*^*p* < 0.05 compared with WT mice; ^#^*p* < 0.05 compared with negative control group.

The effects of MLH3 in patients with gan-shen Yin deficiency syndrome were further explored in MLH3 (−/− and −/+) mice. The results of real-time quantitative PCR showed that the expression of MLH3 mRNA (Figure 1) in patients with gan-shen Yin deficiency was lower than that in patients with non-gan-shen Yin deficiency and ROC analysis confirmed the status of MLH3 in gan-shen Yin deficiency syndrome. As we all know, Liuweidihuang wan is a kind of classical prescription for Yin-Xu. We then examined the effects of Liuweidihuang wan in MLH3 (−/−) mice and MLH3 (−/+) mice with tumor, DM and HBP. The result showed that the levels of cAMP in the plasma of mice (MLH3−/−, −/+) was significantly lower than that in WT mice. And the ratio of E_2_/T in the serum of mice (MLH3−/−, −/+) was significantly higher than that in WT mice (*p* < 0.05). Liuweidihuang wan treatment partly increased the levels of cAMP and decreased the ratio of E_2_/T in MLH3(−/+) mice, but not in MLH3(−/−) mice (Figure 2). However, Liuweidihuang wan had no effects on the size of tumor, hyperglycemia, and hypertension (Figure 1S in Supplementary Material available online at https://doi.org/10.1155/2017/4109828).

## 4. Discussion

The theory of syndrome differentiation plays a prominent role in the diagnosis and the treatment decision of TCM. According to the theory of Yin and Yang, the occurrence, development, and changes of disease lie in the imbalance between Yin and Fang. Clinically all kinds of diseases, including pathological changes of complexion, voice, and pulse condition as well as the nature of diseases, can be generalized and analyzed with the theory of Yin and Yang. [10]. Health is achieved through a balance of Yang and Yin, while Ying deficiency implies a diminishing material level in the physiological functioning of the body. The gan-shen Yin deficiency syndrome is a kind of typical syndrome of Yin deficiency syndrome, for commonly insufficiency essence and blood in the liver and kidney. As we all know, the essence and the blood belong to Yin and are the material basis of Yin. According to our study's findings, 17 mRNAs (*MLH3*,* ACSL6*,* CD55*,* CR1*,* XRCC4*,* CTSK*,* KRAS*,* DDE3B*,* CCNB1*,* PAICS*,* SEC62*,* PSMD6*,* BIRC3*,* TCF7*,* NT6E*,* PSPH*, and* TRCP1*) in PBMC of PLC patients with gan-shen Yin deficiency were different from those in PLC patients without gan-shen Yin deficiency [2].

To further validate that these 17 variables were for gan-shen Yin deficiency syndrome in TCM, but not for PLC disease, DM and HBP patients with the same syndrome of gan-shen Yin deficiency were also included in this study. A total of 244 effective cases of in-patients with PLC, DM, and HBP were collected. The objective clinical manifestations of them from traditional four diagnostic methods were collected by BCQ. Of them, 119 patients were classified as gan-shen Yin deficiency group; others were classified as non-gan-shen Yin deficiency group. No difference of basic characteristics, but BMI, between syndromes of gan-shen Yin deficiency and non-gan-shen Yin deficiency was found. The BMI in the gan-shen Yin deficiency group with DM was significantly lower than that in non-gan-shen Yin deficiency group. This finding might be explained that gan-shen Yin deficiency patients always have the symptom of emaciation, compared to non-gan-shen Yin deficiency patients.

By real-time RT-PCR and ROC analysis, only MLH3 expressions were lower in patients with gan-shen Yin deficiency syndrome than that in patients without gan-shen Yin deficiency syndrome among 17 variables. Other 16 variables might be related to PLC. MLH3 is a MutL homolog protein in mammals. Its basic role is in the DNA mismatch repair mechanism, while it has been proposed to play a distinct role in the meiotic recombination mechanism [11, 12]. Inactivation of the MLH3 gene has been suggested to play a role in both male and female infertility [13]. These findings are, in part, consistent with the symptom of decreased reproductive ability in gan-shen Yin deficiency patients. However, the mechanism of MLH3 in gan-shen Yin deficiency needs to be further explored.

MLH3 KO mice were used to validate the effects of MLH3 in vivo. Reduced activity, decreased and yellow urine, and decreased reproductive ability were found in MLH3−/− and MLH3−/+ mice, compared with WT mice. The symptoms in MLH3−/+ mice, but not in MLH3−/− mice, could be ameliorated by Liuweidihuang wan treatment, which is a classical prescription for gan-shen Yin deficiency. Furthermore, the reduced cAMP and the enhanced ratio of E_2_/T in MLH3−/+ and MLH3−/− mice were found, compared with WT mice, which is consistent with previous reports [14, 15]. It seems that enhanced ratio of E_2_/T might be related to the decreased reproductive ability in male patients with gan-shen Yin deficiency. Liuweidihuang wan could increase the level of cAMP and reduce the ratio of E_2_/T in MLH3−/+ mice. The phenomenon could be explained by the following: (i) MLH3 play a part in the effects of Liuweidihuang wan; as a result, Liuweidihuang wan could not affect the syndrome and the level of cAMP, T, and E2 in MLH3−/− mice; (ii) the symptoms in MLH3−/+ mice were similar to the symptoms in MLH−/− mice, although the expression of MLH3 in MLH3−/+ mice is still retained (Figure 2S). The function of MLH3 might be affected by Liuweidihuang wan in MLH3−/+ mice.

In conclusion, results of this study demonstrate that the MLH3 might contribute to the specific manifestation in patients with gen-shen Yin deficiency. We recommend that future studies explore the relationship between MLH3 and gan-shen Yin deficiency syndrome by expanding the type of disease and the number of samples. Moreover, the mechanism of MLH3 in patients should be conducted to further elucidate the effect of MLH3 in gan-shen Yin deficiency syndrome.

## Supplementary Material

Fig 1S: Liu Wei Di Huang Wan had no effects on the size of tumor, hyperglycemia and hypertention.
Fig 2S: The expression of MLH3 was lower in MLH3−/− mice mice than in WT mice and MLH3−/+ mice.

## Figures and Tables

**Table 1:** Distribution of patients in age, gender, and clinical characteristics.

	Variables	Gan-shen Yin deficiency	Non-gan-shen Yin deficiency	*p* value^*∗*^
PLC	Age (years)			
	≤30	1	0	
	31–50	14	11	*P* = 0.183
	51–70	10	13	
	>70	0	1	
	Gender			
	Male/female	24/1	22/3	*P* = 0.609
	Tumor size			
	≤5 cm	25	25	*P* = 1
	AFP level			
	>400	16	11	*P* = 0.156
	Liver cirrhosis			
	Yes	17	13	*P* = 0.248
	No	8	12	

HBP	Age (years)			
	≤40	2	1	
	41–80	41	47	*P* = 0.416
	>80	1	2	
	Gender			
	Male/female	30/14	34/16	*P* = 0.985
	Duration of HBP (years)			
	<5	12	13	
	5–10	23	24	
	11–20	6	6	*P* = 0.580
	>20	3	7	
	Classification of hypertension			
	Class I	5	3	
	Class II	28	33	*P* = 0.508
	Class III	11	14	

DM	Age (years)			
	<40	8	9	
	40–60	24	21	
	61–80	14	16	*P* = 0.871
	>80	4	4	
	Gender			
	Male/female	36/14	35/15	*P* = 0.826
	Duration of DM (years)			
	<5	24	21	
	5–10	12	13	
	11–20	10	12	*P* = 0.577
	>20	4	4	
	BMI (Kg/m^2^)	23.57 ± 2.97^*∗*^	26.44 ± 4.14	*P* = 0.001

^*∗*^ *p* < 0.01 compared with non-gan-shen Yin deficiency group.
