# Supplementary material for: Reduced MLH3 Expression in the Syndrome of Gan-Shen Yin Deficiency in Patients with Different Diseases
Source: Evid Based Complement Alternat Med. 2017 Sep 26;2017:4109828. doi: 10.1155/2017/4109828 (PMC5634572; doi:10.1155/2017/4109828)
Supplement: Supplementary file 1 — Fig 1S: Liu Wei Di Huang Wan had no effects on the size of tumor, hyperglycemia and hypertention. Fig 2S: The expression of MLH3 was lower in MLH3−/− mice mice than in WT mice and MLH3−/+ mice. [file 4109828.f1.pdf]

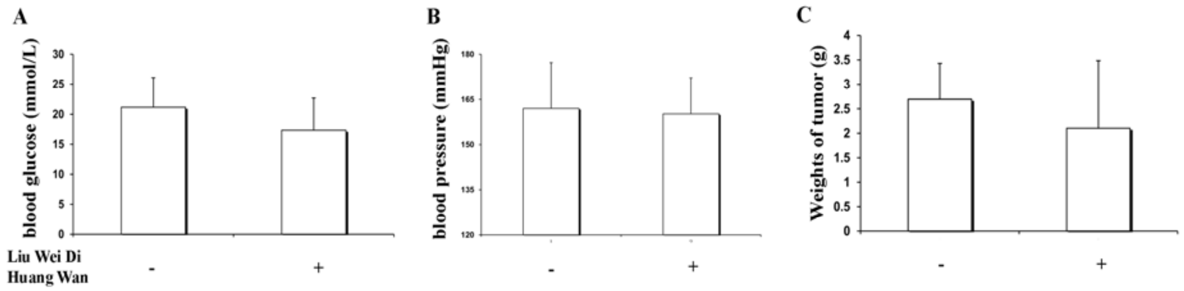

Fig. 1S Liu Wei Di Huang Wan had no effects on the hyperglycemia (A), the hypertension (B) and the size of tumor (C) in *MLH3*(-/+) mice with hyperglycemia, hypertension and tumor, separately.

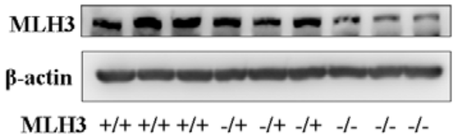

Fig. 2S The expression of MLH3 in WT mice, *MLH3*-/+ mice and *MLH3*-/- mice. The expression of MLH3 was detected by western-blot.
